# Tissue Context Shapes Distinct Premalignant Outcomes in an HPV16 E6/E7-Mutant Pik3ca Transgenic Mouse Model

**DOI:** 10.1158/2767-9764.CRC-25-0789

**Published:** 2026-07-22

**Authors:** Marina Ayre, Gonzalo Fernandez-Ugazio, Anabel V. DiGaudio, Omar Adrián Coso, J. Silvio Gutkind, Ana Rosa Raimondi

**Affiliations:** 1 Instituto de Fisiología, Biología Molecular y Neurociencias (IFIBYNE-UBA-CONICET), Consejo Nacional de Investigaciones Científicas y Técnicas, Buenos Aires, Argentina.; 2 Argentina Consortium for Research and Training in Virally Induced AIDS Malignancies, University of Miami- Center for AIDS Research (UM-CFAR)/Sylvester Comprehensive Cancer Center (CCC), University of Miami Miller School of Medicine, Miami, Florida.; 3 Departamento de Patología, Hospital Zubizarreta, Buenos Aires, Argentina.; 4 Departamento de Fisiología, Biología Molecular y Celular, Facultad de Ciencias Exactas y Naturales, Universidad de Buenos Aires, Buenos Aires, Argentina.; 5 Gleiberman Head and Neck Cancer Center, Moores Cancer Center, University of California San Diego Health, La Jolla, California.; 6 Department of Pharmacology, University of California San Diego, School of Medicine, La Jolla, California.; 7 Facultad de Odontología, Cátedra de Anatomía Patológica, Universidad de Buenos Aires, Buenos Aires, Argentina.

## Abstract

High-risk human papillomavirus (HPV) infection, particularly HPV16, is a major etiologic factor associated with multiple human cancers, including anal, cervical, and oropharyngeal carcinomas. However, the mechanisms underlying tissue-specific responses to HPV oncogene expression remain poorly understood. In this study, we developed and characterized a double-inducible transgenic mouse model enabling spatially and temporally controlled expression of HPV16 E6/E7 oncoproteins in K14-positive epithelial progenitor cells of both the anal and oral mucosae. Within 2 months of induction, HPV16 E6/E7 expression elicited epithelial hyperplasia and dysplasia in both tissues but with distinct outcomes: low-grade squamous intraepithelial lesions in the anal canal and moderate to severe dysplasia in the tongue. Continuous oncogene expression was required for lesion maintenance, as doxycycline withdrawal reversed the proliferative phenotype, indicating a dynamic and reversible process. To assess whether activation of the PI3K pathway could cooperate with HPV oncogenes, we combined the E6/E7 model with a *Pik3ca*^H1047R^ knock-in mouse line. Constitutive PI3K activation alone induced mild dysplasia in both epithelia, whereas its combination with E6/E7 enhanced dysplastic severity in the tongue but not in the anal mucosa. No progression to invasive carcinoma was observed within 6 months. These findings demonstrate that identical oncogenic signals—HPV16 E6/E7 expression and PI3K activation—produce distinct premalignant outcomes depending on epithelial context. This work highlights the critical role of local microenvironmental factors in HPV-driven carcinogenesis and provides a platform to identify tissue-specific therapeutic vulnerabilities in HPV-associated cancers.

**Significance:**

This work presents a double-inducible mouse model showing that HPV16 E6/E7 and PI3K activation drives distinct dysplastic outcomes in anal and oral epithelia. The study highlights how tissue context and microenvironment critically shape HPV oncogene effects, offering an important tool for uncovering mechanisms and therapeutic targets in HPV-related cancers.

## Introduction

Infection with high-risk subtypes of human papillomavirus (HPV), primarily HPV16 and HPV18, is associated with the development of squamous cell carcinoma (SCC) of the cervix, anus, vulva, penis, and head and neck (1, 2). In recent decades, there has been an increase in the incidence of oropharyngeal SCC and anal SCC (ASCC), both related to HPV infection (3–5). Conversely, the incidence of cervical cancer linked to HPV has already shown a marked decline, largely attributable to the widespread adoption of routine screening and the development of HPV vaccines (6–9). HPV vaccination has the potential to significantly alter the epidemiology of all HPV-associated SCCs. Nonetheless, vaccination coverage remains suboptimal, which hinders efforts to reduce viral transmission and, ultimately, to lower the incidence of these cancers (3–5, 8).

To fully understand the development of HPV-associated cancers, it is necessary to examine in detail the different epithelial sites where viral gene expression becomes deregulated (10). Although HPV has been extensively studied in malignancies of the female genital tract, relatively little is known about the specific pathogenesis of ASCC and head and neck SCC (HNSCC; refs. 10, 11).

In this regard, genetically engineered mouse models are powerful tools to investigate specific cause and effect relationships between molecular changes and tumor initiation and progression. However, the use of genetically defined approaches to study anal or oral carcinogenesis by HPV is very limited (12–14). For example, expression of the *E6* and *E7* oncogenes in the anal epithelium under the control of the constitutive cytokeratin (K) 14 promoter leads to SCC development only upon chemical carcinogen treatment (15), likely not reflecting the natural evolution of ASCC. Furthermore, transgenic mice carrying the HPV16 early region under the control of the K14 constitutive promoter developed epidermal and cervical lesions, as well as SCC at the base of the tongue after 30 weeks (16, 17). To date, no study has simultaneously and comparatively assessed the effects of *E6* and *E7* oncogenes in anal and oral squamous epithelia to define their site-specific oncogenic impact.

For this purpose, we used our previously developed K14-CreER^tam^ transgenic mouse system, which targets the basal layer of the oral squamous epithelium via tamoxifen (TAM)-inducible Cre recombinase under the K14 promoter (18). In the present study, we extended its use to the anal epithelium by characterizing the recombination pattern in this tissue. We then combined this model with our previously established doxycycline (DOX)-inducible E6/E7 system (19), enabling conditional HPV oncogene expression in K14-positive cells. This dual system provides a versatile model to compare site-specific effects of E6/E7 expression in anal and oral squamous epithelia. Hence, we demonstrate that the expression of HPV16 E6/E7 (TG-E6/E7) in an epithelial compartment that includes the anal and oral progenitor cells, using a double-inducible control system, results in the development of dysplastic changes within 2 months in both tissues.

Phosphatidylinositol-4,5-bisphosphate 3-kinase catalytic subunit alpha (*PIK3CA*), encoding the catalytic subunit of PI3Kα, is the most frequently mutated oncogene in HNSCC (∼20%) and is enriched in HPV-positive tumors (25%; refs. 20, 21). Also, mutations in *PIK3CA* are found in approximately 20% of human anal cancers (22). Three common hotspot mutations in the *Pik3ca* gene—E542K and E545K in exon 9 within the helical domain and H1047R/L in exon 20 within the kinase domain of p110α—result in constitutive PI3K pathway activation that occurs independently of the p85 regulatory subunit (23). H1047R substitution is the most common mutation in the kinase domain, leading to the constitutive activation of PI3K signaling independent of the PI3K regulatory subunit p85 (24, 25). Based on this, we evaluated whether TG-E6/E7 lesions could progress through PI3K pathway activation. By breeding TG-E6/E7 mice with a *Pik3ca*^H1047R^ transgenic line, we were able to assess the differential role of mutated *Pik3ca* in both tissues studied. The compound mice (C.M.) rapidly developed a significantly higher incidence of dysplasia in the tongue than in the anal mucosa, which only exhibited changes consistent with low-grade squamous intraepithelial lesions (LSIL). These findings demonstrate that identical signaling alterations yield distinct premalignant outcomes depending on the tissue context in which HPV16 E6/E7 are expressed. Moreover, the use of this validated TG-E6/E7 animal model may help recapitulate the progression of anal and oral carcinogenesis induced by HPV in a genetically defined system, thereby facilitating the study of the molecular mechanisms driving these processes in two anatomic sites that are critical for understanding HPV-related carcinogenesis.

## Materials and Methods

### Transgenic mice

The K14-CreER^tam^, Ai14, Tet-E6/E7, Rosa26-rtTA-IRES-EGFP-rtTA^flox/flox^, *Pik3ca*^H1047R^ mouse strains have been described previously (18, 19, 26–28). All mice were maintained under a 12:12-hour dark/light cycle with food and water provided *ad libitum*. They were grouped-housed (3–5 mice per cage). Male and female mice were used and randomly assigned to the different experimental groups. All mice were euthanized by carbon dioxide inhalation using appropriate equipment provided by the animal facility.

### Transgenic mice models

Rosa26-rtTA-IRES-EGFP-rtTA^flox/flox^ mice were crossed with Tet-E6/E7 mice to generate Rosa26-rtTA-IRES-EGFP-rtTA^+/flox^/Tet-E6/E7 and subsequently bred with the K14-CreER^tam^ line to generate K14-CreER^tam^/Rosa26-rtTA-IRES-EGFP-rtTA^+/flox^/Tet-E6/E7 (herein referred to as TG-E6/E7; Supplementary Fig. S1A). These mice were further crossed with *Pik3ca*^H1047R/+^ to generate K14-CreER^tam^/Rosa26-rtTA-IRES-EGFP-rtTA^+/flox^/Tet-E6/E7/*Pik3ca*^H1047R/+^ (C.M.; Supplementary Fig. S1B). The K14-CreER^tam^ driver line was crossed with the *Pik3ca*^H1047R^ mouse line to generate K14-CreER^tam^/*Pik3ca*^H1047R/+^ (herein referred to as K14/PI3K) and with the Ai14 (Rosa-LSL-tdTomato) reporter line to obtain K14-CreER^tam^/Ai14 mice. Littermate animals with intermediate genotypes or lacking induction were used as controls, depending on the specific experimental design. All mice used for the experiments were on a mixed FVB/N–C57BL/6 genetic background. Mice were maintained in accordance with the guidelines of the Animal Facility of the School of Sciences of the University of Buenos Aires (UBA) and government regulations (Servicio Nacional de Sanidad y Calidad Agroalimentaria, RS617/2002, Argentina). All procedures were approved by the Institutional Animal Care and Use Committee (Protocol # 90/2017 and 90b/2022) of the School of Sciences of the UBA. All efforts were made to minimize the number of animals used and their suffering.

TAM (Sigma) was administered to 1-month-old animals at 1 mg per mouse per day orally for five consecutive days (18). DOX was then administered in the drinking water at a concentration of 2 g/L (Santa Cruz Biotechnology, sc-204734C) *ad libitum* throughout the experimental period indicated in each case (Supplementary Fig. S1A and S1B). All mice were examined daily. Genotyping was performed on tail biopsies by PCR using specific primers (Supplementary Table S1).

### Histology, immunohistochemistry, and immunofluorescence

Two hours before euthanasia, mice were, in some cases, injected intraperitoneally with 5′-bromo-2′-deoxyuridine (BrdUrd) at 100 μg/g body weight for cell proliferation detection. Tissues were dissected, fixed overnight in buffered 4% paraformaldehyde (PFA) at room temperature, dehydrated, and embedded in paraffin for routine histopathologic analysis. Hematoxylin and eosin–stained sections were used for diagnostic purposes and unstained serial sections for immunostaining. Immunohistochemistry (IHC) was performed as previously described (29). Immunofluorescence (IF) was performed on the cryostat section from snap-frozen optimal cutting temperature (OCT)–embedded tissue samples. The tissues were fixed with 4% PFA (3 hours) and subsequently were kept in a 30% sucrose solution for 48 hours. They were then sectioned and embedded in OCT embedding medium (Sakura Inc.). Once the OCT blocks were prepared and solidified, they were immediately stored at −80°C until histologic sectioning in a cryostat at −20°C. Tissues were blocked with a solution composed of 10% goat serum, 2% BSA, and 0.15% Triton X-100 (30). An Alexa Fluor 488 (Thermo Fisher Scientific)–conjugated secondary antibody was used for detection of enhanced green fluorescent protein (EGFP). The slides were counterstained with 4′,6-diamidino-2-phenylindole and mounted with VECTASHIELD. They were then stored at 4°C and protected from light until images were captured via confocal microscopy. Antibodies used include BrdUrd (IIB5) 1:400 from Santa Cruz Technology, E6 (1:100 and 1:150) and K7 (1:700) from Abcam, EGFP (1:3,000) and phospho-S6 (pS6; 1:100) from Cell Signaling Technology, and K14 (1:1,000) from BioLegend. Images were acquired using Zeiss LSM 900 and Zeiss Apotome microscopes [Instituto de Fisiología, Biología Molecular y Neurociencias (IFIBYNE) and IFIBIO Houssay facilities].

### pS6 quantification

pS6-immunostained histologic slides containing tongue and anal mucosa samples from 4 to 5 mice per group were scanned, digitized, and subjected to unbiased cell detection and automated quantification using the digital pathology platform QuPath (version 0.4.4, https://qupath.github.io; ref. 31), similar to our previous analyses (32). Briefly, 2 to 3 independent 20× images were acquired using an Axio Imager microscope and stored as TIFF files at 0.322 μm/pixel. Images were then imported into QuPath, in which a region of interest encompassing the entire epithelium was delineated (mean area analyzed per section: anal mucosa, 38,903 μm^2^; tongue, 43,502 μm^2^). Automatic color deconvolution was applied to separate 3,3′-Diaminobenzidine (DAB) and hematoxylin signals. Cell and nucleus segmentation was performed using the optical density sum (σ = 1–1.5 μm; minimum cell size = 10 μm^2^; maximum cell size = 600 μm^2^). Positive cells were automatically identified using mean cytoplasmic DAB optical density with a 0.1 threshold above background, ensuring unbiased detection. The number of positive cells was normalized to the total number of epithelial cells (percentage) and to epithelial area per section (density). At least 250 cells were analyzed per image. The average per animal was calculated from all available images, and statistical analyses were performed using one-way ANOVA per animal.

### Statistical analysis

Data are presented as frequency distribution. The Fisher exact test was selected to compare differences between two groups. For analysis of categorical variables with more than one factor, we first conducted an omnibus log-linear analysis for an A × B × C contingency table, followed by uncorrected Fisher exact tests for *post hoc* pairwise comparisons using an online calculator (33) http://www.vassarstats.net/index.html. All tests conducted were two-sided and considered statistically significant at *P* ≤ 0.05.

## Results

### The K14-CreER^tam^ expression system enabled Cre-mediated recombination in the anal and oral mucosae

**Figure 1.**
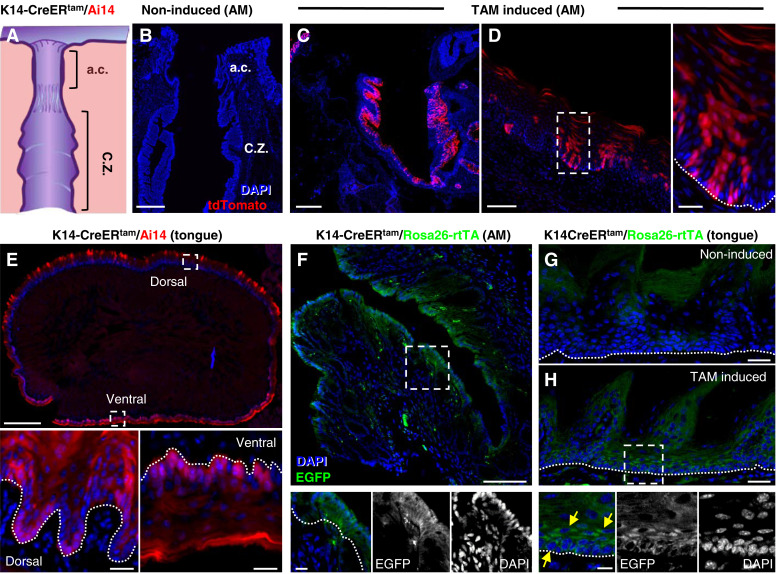
The K14-CreER^tam^ expression system enabled the Cre-mediated recombination in the anal and oral mucosae. **A,** Schematic representation of the anal canal. **B,** Anal mucosa (AM) of an uninduced K14-CreER^tam^/Ai14 mice (reporter animal) without tdTomato expression at low magnification. a.c., anal canal; C.Z., colorectal zone. **C** and **D,** Administration of TAM to reporter animals induces the expression of tdTomato in epithelial cells along the mucosal lining of the anal canal. **D,** AM from a reporter mice after 1 month of TAM treatment. Right, region outlined by a dashed rectangle is shown at higher magnification. The right inset shows tdTomato-positive cells extended from the basal layer into the superficial layers, forming clusters and columns of labeled cells 1 month after induction. **E,** Representative sagittal tongue section from a reporter mouse showing tdTomato expression after 1 month of induction. White dashed squares indicate the dorsal tongue and the ventral zone, respectively. Both squares are shown below at higher magnification. tdTomato-positive cells extended from the basal layer into the superficial layers, forming clusters and columns of labeled cells 1 month after induction. **F,** Anal canal from K14-CreER^tam^/Rosa26-rtTA-IRES-EGFP-rtTA^flox^ animals (K14-CreER^tam^/Rosa26-rtTA); EGFP (green) expression in epithelial cells of the mucosal lining of the anal canal following TAM administration. The area outlined by a dashed rectangle is shown at higher magnification below. **G** and **H,** Tongue from K14-CreER^tam^/Rosa26-rtTA mice with and without TAM treatment. **H,** The area outlined by a dashed rectangle is shown at higher magnification below. Counterstaining with 4′,6-diamidino-2-phenylindole (DAPI) for nuclear localization (blue fluorescence). Scale bars, 1 mm (**B** and **C**); 500 μm (**E**); 100 μm (**D** and **F**); 25 μm (**G** and **H** and insets in **D–F**); 10 μm (insets in **G** and **H**).

To investigate the effects of HPV16 E6/E7 expression simultaneously in the anal canal and tongue, we used the K14-CreER^tam^ transgenic mouse line, which expresses a TAM-inducible Cre recombinase capable of mediating LoxP-dependent recombination in the basal layer of the oral epithelium (18) and the anal canal epithelium, as we demonstrated here. To confirm the specificity of Cre-dependent recombination as well as the expression of the K14-Cre line in the anal epithelium, we crossed the aforementioned driver line with the Ai14 (Rosa-LSL-tdTomato) reporter line. Oral administration of TAM to K14-CreER^tam^/Ai14 mice resulted in tdTomato expression in epithelial cells along the epithelium lining of the anal canal and in epithelial cells of the tongue, with no “leaky” expression observed in non-induced animals (Fig. 1A and B). As predicted, tdTomato-positive cells extended from the basal layer into the superficial layers, forming clusters and columns of labeled cells 1 month after induction, both in the tongue (as expected from 18) and in the anal mucosa (Fig. 1C–E). The presence of labeled cell clusters and labeling retention for over a month in these two rapidly renewing epithelia supports the Cre-mediated targeting of epithelial progenitor cells by this mouse line. Although no tdTomato labeling was observed in solvent-treated mice, we decided to further increase HPV16 E6/E7 selectivity using a double-inducible control system. For this purpose, K14-CreER^tam^ mice were crossed with Rosa26-rtTA-IRES-EGFP^flox^ mice to subsequently achieve E6/E7 expression using a Tet-E6/E7 transgenic line (19, 27). In these triple transgenic mice, Cre-mediated recombination upon TAM induction renders targeted cells susceptible to DOX-mediated induction, enabling exceptional cellular and temporal control of the E6/E7 expression (Supplementary Fig. S1A). Finally, we confirmed the tissue-specific nature and the efficient temporal control capability of the driver system with the Rosa26-rtTA reporter line for the murine anal canal and tongue by EGFP expression (Fig. 1F–H).

### The expression of E6/E7 in the mucosa of the anal canal produces LSIL and high-grade squamous intraepithelial lesions

**Figure 2.**
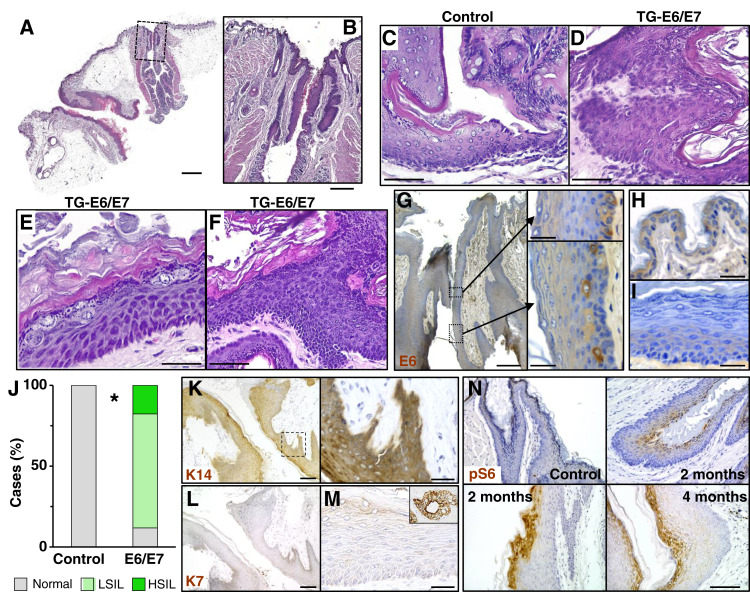
E6/E7 expression is sufficient to induce the development of squamous intraepithelial lesions in the anal canal of TG-E6/E7 mice. **A,** Panoramic view at low magnification of the genital area in a female mouse, showing the location of the murine anal canal (black dotted rectangle) in relation to the vagina (bottom left). **B,** Higher magnification of the area outlined in the rectangle in **A**, showing the normal histology of the anal canal in a control animal. **C–F,** Morphologic changes developed in the TG-E6/E7 anal mucosa. **C,** Representative histologic image of the anal mucosal epithelium from a control mouse. **D–F,** Representative examples of LSIL in TG-E6/E7 anal mucosa (AM). **D,** Anal mucosa lined by the hyperorthokeratotic squamous epithelium showing mild to moderate dysplastic changes 2 months after DOX treatment. **E,** AM with moderate dysplastic changes after 2 months of evolution. Note the hypergranulosis, basal stratification, and cellular and nuclear irregularities involving the lower and middle epithelial layers. **F,** AM with severe dysplastic changes (HSIL) after 4 months of evolution. Hyperkeratosis and cellular and nuclear irregularities involving the middle and upper epithelial layers. **G,** IHC expression of E6 in the anal mucosa of TG-E6/E7 mice. In **G**, a low-magnification image shows the patchy pattern of E6 expression along the anal canal. Black rectangles indicate areas with positive staining, shown at higher magnification to the right. Note the expression of E6 in the basal epithelial layer (top) as well as the cytoplasmic localization of E6 in individual suprabasal cells (bottom). **H,** Perianal skin from the E6/E7 transgenic mouse shown in **G**, lacking E6-positive cells. **I,** Control without primary antibody in TG-E6/E7 anal mucosa. **J,** Incidence of LSIL and HSIL in the anal mucosa of control mice (*N* = 10) and TG-E6/E7 animals (*N* = 17) as the percentage of the total number of animals per group. LSIL, cases diagnosed with mild dysplasia, hyperplasia, and cellular and tissue alterations involving the lower third of the epithelium; HSIL, diagnosed with moderate or severe dysplasia. TG-E6/E7: 2 months (*N* = 7), 4 months (*N* = 5), and 5–6 months (*N* = 5). *, *P* < 0.05 by Fisher exact test. **K,** IHC expression of K14. Low-magnification overview of TG-E6/E7 anal mucosa showing K14 expression. Right, a higher-magnification image of a hyperplastic and hyperkeratotic area shows K14 expression throughout the entire epithelial thickness. **L,** Immunostaining for K7. Low-magnification overview of TG-E6/E7 anal mucosa showing no K7 expression. **M,** Detailed view of an TG-E6/E7 anal mucosal area negative for K7. Inset, positive control for K7; glandular epithelium (intestine). **N,** Immunostaining for pS6 in the anal mucosa of TG-E6/E7 animals. Anal mucosa of a control animal showing minimal pS6 expression. pS6 expression in anal mucosa 2 months after E6/E7 induction, observed in the suprabasal layers; pS6 expression in anal mucosa of an animal 4 months after E6/E7 induction. Scale bars, 1 mm (**A**); 250 μm (**B**); 200 μm (**C**); 100 μm (**K** and **L**); 50 μm (**F–I** and **N**); 25 μm (**D**, **E**, **M**, and insets in **C** and **K**).

Within 2 months following the induction of HPV16 E6/E7 expression in triple transgenic (TG-E6/E7) mice (Fig. 2), histopathologic analysis revealed hyperplastic and dysplastic alterations in the anal mucosa in 85% of the cases examined (6/7), with diagnoses ranging from mild to moderate dysplasia (LSIL; Fig. 2A–E). Alongside histologic characterization of the TG-E6/E7 anal epithelium, IHC analysis confirmed E6 expression. E6-positive cells were detected as isolated cells in the basal and suprabasal layers and as small suprabasal clusters distributed along the anal canal epithelium (Fig. 2G). We next investigated whether the LSIL in TG-E6/E7 mice progressed over time by extending the period of *E6/E7* oncogene induction to 4 months (Supplementary Fig. S2). Four months of DOX exposure produced cytologic changes similar to those seen after 2 months, with only one of five cases progressing to high-grade squamous intraepithelial lesions (HSIL; severe dysplasia; Fig. 2F). At 6 months, lesions persisted without progression. As LSILs showed minimal progression after 2 months of E6/E7 induction, another group of mice underwent 2 months of DOX treatment followed by a 2-month withdrawal to assess the need for sustained expression (Supplementary Fig. S2). After 4 months, no dysplasia was present, and mild hyperkeratosis with reduced basal proliferation was observed (3/3; Supplementary Fig. S3). BrdUrd labeling confirmed decreased cell proliferation after DOX withdrawal, indicating that the proliferative phenotype depends on sustained E6/E7 expression and that lesions do not progress without it (Supplementary Fig. S4). Then, we analyzed the expression of pS6, a downstream marker of mTOR pathway activation, in LSILs after 2 months of induction, as its accumulation has been reported in several HPV-associated cancers—including HNSCC, cervical carcinoma, and ASCC—suggesting that mTOR activation may play a role in their development (15, 34, 35). pS6 expression was observed in the suprabasal layer of the TG-E6/E7 anal epithelium, in clear contrast to the control anal mucosa, which showed minimal pS6 expression (Fig. 2N). Moreover, unbiased quantification of pS6-labeled cells using QuPath software revealed a statistically significant increase in pS6-positive cells following E6/E7 overexpression, as reflected both in the percentage of positive cells relative to the total epithelial cell population and in their density per unit epithelial area (Supplementary Fig. S5). Additionally, we found no expression of K7, a marker highly expressed in the squamocolumnar epithelium, whereas K14, a marker highly expressed in the squamous epithelium, showed widespread staining (Fig. 2K–M). Overall, histopathologic analysis revealed that LSIL was highly prevalent in TG-E6/E7 mice, with a small proportion of mice displaying more severe lesions (Fig. 2J). The proliferative phenotype and LSILs observed in TG-E6/E7 animals are most likely consequences of sustained E6/E7 expression.

### The expression of E6/E7 in the lingual mucosa produces dysplastic lesions

**Figure 3.**
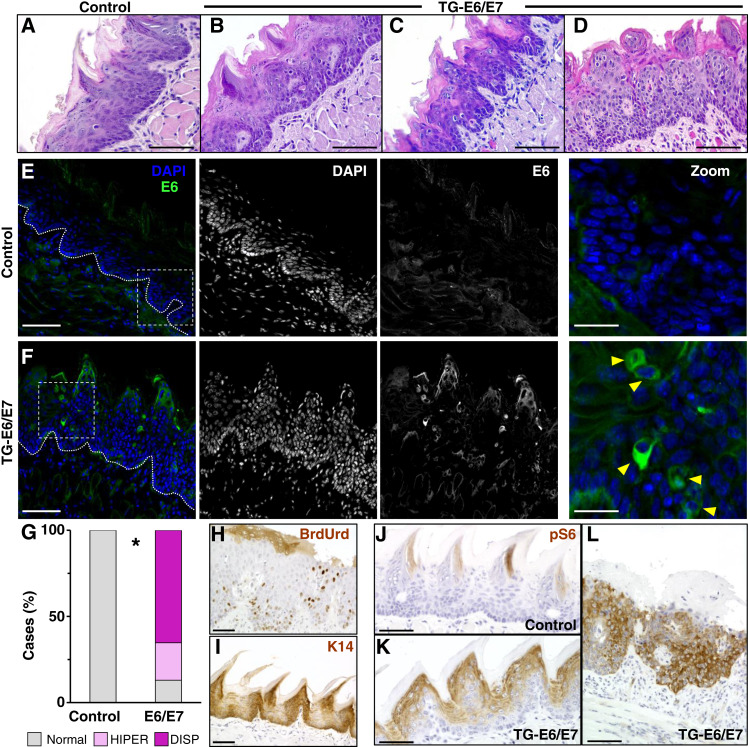
Development of dysplastic lesions in the tongue epithelium upon E6/E7 expression. **A–D,** Morphologic changes developed in the TG-E6/E7 lingual mucosa. **A,** Histologic image of the control murine lingual epithelium. **B,** Representative image of an area of the lingual epithelium with mild dysplastic changes 2 months after E6/E7 induction. **C,** Area of the transgenic tongue epithelium showing moderate dysplasia without marked hyperplasia. **D,** Transgenic tongue showing histologic changes consistent with severe epithelial dysplasia 4 months after DOX treatment. **E** and **F,** Expression of E6 in the transgenic E6/E7 lingual epithelium. Representative images of immunostaining for E6 (green fluorescence) in a control tongue (**E**) and in a tongue from a TG-E6/E7 animal (**F**). Yellow arrow heads indicate epithelial cells expressing E6. Counterstaining with 4′,6-diamidino-2-phenylindole (DAPI) for nuclear localization (blue fluorescence). **G,** Incidence of hyperplasia and dysplasia in the tongue epithelium of control mice (*N* = 10) and TG-E6/E7 animals (*N* = 23) as the percentage of total number of animals per group. DISP, dysplasia; HIPER, hyperplasia (); diagnosed with mild, moderate, or severe dysplasia. TG-E6/E7: 2 months (*N* = 11), 4 months (*N* = 6), and 5–6 months (*N* = 6). *, *P* < 0.0001 by Fisher exact test. **H,** Positive immunostaining for BrdUrd in the lingual epithelium of a TG-E6/E7 animal with 4 months of evolution. **I,** K14 expression from the basal layer to the suprabasal strata in the lingual epithelium after 4 months of evolution. **J,** Tongue from a control animal showing minimal pS6 expression. **K,** pS6 expression in the upper layers of the epithelium 2 months after E6/E7 expression. **L,** Widespread pS6 expression throughout the epithelium 4 months after E6/E7 induction. Scale bars, 50 μm (**A** and **C–L**); 25 μm (zoomed views of **A** and **B**).

Comparative morphologic analysis of TG-E6/E7 tongues revealed cellular and nuclear irregularities in the lower layers of the lingual epithelium (Fig. 3), with areas of hyperplasia (18% 2/11) and a larger proportion of tongues with regions diagnosed with dysplasia (64%, 7/11) after 2 months of follow-up (Fig. 3B and C). Dysplasia varied in severity from mild to severe. None of the histologic changes observed in the tongue were present in the control group (Fig. 3A). After 4 months of follow-up, 100% of transgenic tongues (5/5) showed moderate to severe dysplasia (Fig. 3D). At 6 months, lesions persisted without progression. Furthermore, similar to the anal samples, none of the TG-E6/E7 tongues exhibited morphologic alterations 2 months after DOX withdrawal (3/3; Supplementary Fig. S3B). E6 expression in TG-E6/E7 tongues was assessed using IF, revealing scattered E6-positive cells in the layer just above the basal compartment and small clusters of suprabasal cells along the tongue epithelium, with a predominantly cytoplasmic staining pattern (Fig. 3E and F). With regard to mTOR activity at 2 months of progression, pS6 staining revealed an increased number of positive cells in the suprabasal layers (Fig. 3K), which was statistically higher compared with controls (Fig. 3J; Supplementary Fig. S5), whereas the severe dysplastic lesion after 4 months of progression showed staining extending from the basal to the suprabasal layers (Fig. 3L). Furthermore, dysplastic TG-E6/E7 epithelium exhibited increased cell proliferation, accompanied by an extended and intense expression of K14 spanning from hyperplastic to dysplastic regions (Fig. 3H and I). Overall, histopathologic analysis revealed dysplasia in 65% of TG-E6/E7 mice, hyperplasia in 21%, and normal mucosa in the remaining 14% (Fig. 3G). Thus far, we found that the premalignant lesions in the TG-E6/E7 tongue arose earlier and were more severe than those in the transgenic anal mucosa, yet, like the anal mucosa, they were dependent on E6/E7 expression.

### *Pik3ca* ^H1047R^ produces different premalignant outcomes depending on the mucosa affected upon E6/E7 expression

**Figure 4.**
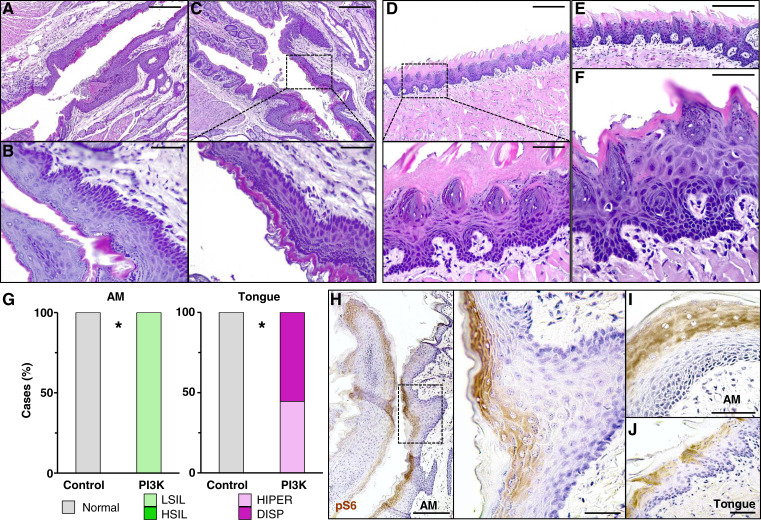
Morphologic changes developed in the anal and lingual mucosae of K14/PI3K animals. Hematoxylin and eosin (H&E)–stained sections of the anal mucosa (AM) showing epithelial alterations at 2 and 4 months following transgene activation. **A,** AM from TG-E6/E7 mice displaying stratified squamous epithelium with hyperkeratosis, hypergranulosis, and areas of basal layer stratification (low magnification, 2 months). **B,** Region of AM exhibiting mild epithelial dysplasia. **C,** At 4 months, transgenic AM shows squamous epithelium with hyperkeratosis, epithelial hyperplasia, and mild dysplastic changes (low magnification). Inset, higher magnification of the area in **C** highlighting epithelial hyperplasia and hyperkeratosis. H&E-stained sections of tongue mucosa showing epithelial alterations at 2 and 4 months after transgene activation. **D,** Low-magnification view of TG-E6/E7 lingual epithelium showing marked hyperkeratosis. Bottom, a higher-magnification image reveals hyperplastic epithelium with cellular and nuclear irregularities in the basal layers and suprabasal hyperkeratosis (2 months). **E,** Low-magnification view of transgenic tongue epithelium showing hyperkeratosis and mild dysplasia (4 months). **F,** Area of a K14/PI3K transgenic tongue displaying mild dysplasia. **G,** Incidence of LSIL and HSIL in the AM of control mice (*N* = 10) and K14/PI3K animals (*N* = 7) as the percentage of the total number of animals per group. LSIL, cases diagnosed with mild dysplasia, hyperplasia, and cellular and tissue alterations involving the lower third of the epithelium; HSIL, diagnosed with moderate or severe dysplasia. Incidence of hyperplasia and dysplasia in the tongue epithelium of control mice (*N* = 10) and TG-E6/E7 animals (*N* = 9) as the percentage of the total number of animals per group. DISP, dysplasia; HIPER, hyperplasia(); diagnosed with mild, moderate, or severe dysplasia. *, *P* < 0.0001 by Fisher exact test. **H,** pS6 expression pattern in the anal canal of K14/PI3K mice (low magnification). Right, a higher-magnification view of the highlighted rectangular area in **H** shows pS6-positive cells in the suprabasal layers. **I,** Another area of K14/PI3K AM displaying dysplastic changes and pS6 immunoreactivity. **J,** Lingual epithelium from a K14/PI3K animal showing pS6 expression in the upper layers. Scale bars, 200 μm (**A**, **C–F**, and **H**); 50 μm (**B**, **I**, **J**, and insets in **C**, **D**, and **H**).

**Figure 5.**
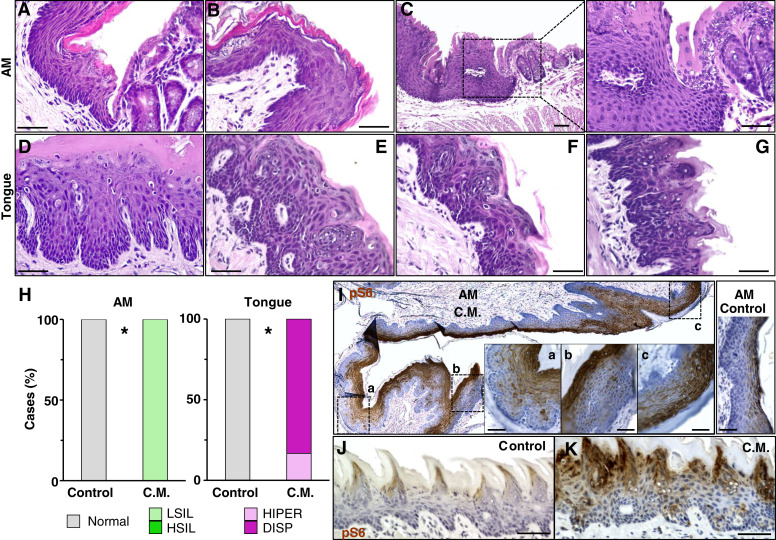
*Pik3ca* ^H1047R^ produces different premalignant outcomes depending on the mucosa affected upon E6/E7 expression. Morphologic changes developed in the anal mucosa (AM) of C.M. **A** and **B,** After 2 months, epithelial hyperplasia and hyperkeratosis were observed, along with focal cellular alterations. Representative low- and high-magnification images. **C,** At 4 months, an area of the AM exhibiting basal layer duplication, epithelial hyperplasia, and hyperorthokeratosis/parakeratosis. Right, a higher-magnification image of the inset in **C**. Morphologic changes developed in the tongue mucosa of C.M. **D** and **E,** Representative images of compound tongues after 2 months of follow-up showing severe (**D**) and moderate (**E**) dysplasia. **F** and **G,** Representative images of severe dysplasia after 4 months of evolution. **H,** Incidence of LSIL and HSIL in the AM of control mice (*N* = 10) and C.M. (*N* = 8) as the percentage of the total number of animals per group. LSIL, cases diagnosed with mild dysplasia, hyperplasia, and cellular and tissue alterations involving the lower third of the epithelium; HSIL, diagnosed with moderate or severe dysplasia. Incidence of hyperplasia and dysplasia in the tongue epithelium of control mice (*N* = 10) and C.M. (*N* = 12) as the percentage of the total number of animals per group. DISP, dysplasia; HIPER, hyperplasia; diagnosed with mild, moderate, or severe dysplasia. *, *P* < 0.0001 by Fisher exact test. **I,** Panoramic view of the AM from a C.M. after 2 months, showing pS6 expression throughout the mucosa. Insets (a–c), higher-magnification images of the boxed regions in **I**. Right, pS6 expression in control AM. **J,** Tongue from a control mouse showing minimal pS6 expression. **K,** Increased expression of pS6 throughout the tongue from a C.M. Scale bars, 50 μm for all panels.

**Figure 6.**
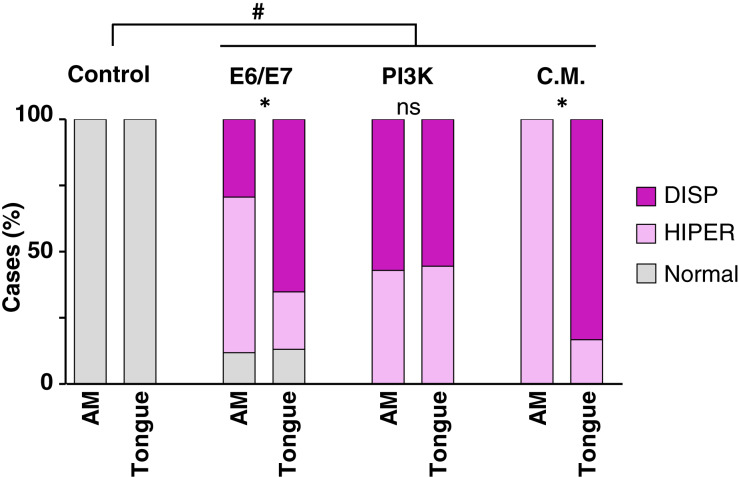
Incidence of hyperplastic and dysplastic lesions in AM and tongue epithelia across all three experimental groups. Incidence was evaluated for all cases analyzed in the TG-E6/E7, K14/PI3K, and C.M. genotypes in both the anal mucosa (AM) and lingual mucosa (tongue). For statistical comparison between the two anatomic sites, cases in the AM were classified as the hyperplasia (HIPER) category including all lesions exhibiting hyperkeratosis, epithelial hyperplasia, or mild dysplasia (LSIL), whereas the dysplasia (DISP) category comprising lesions with a histopathologic diagnosis of moderate to severe dysplasia (HSIL). In the lingual mucosa, the HIPER category included lesions characterized by hyperkeratosis and epithelial hyperplasia, whereas the DISP category encompassed all dysplastic lesions, ranging from mild to severe. The normal category included histologically normal mucosa with morphology within normal limits. Significant interaction among location × histological type × genotype was observed by global log-linear three-way contingency table analysis [G^2^_(17)_ = 148.9; *P* < 0.0001], with a significant histologic type × location interaction [G^2^_(2)_ = 13.9, *P* < 0.001]. #, *P* < 0.001 by Fisher exact test vs. control; *, *P* < 0.005 by Fisher’s exact test in AM vs. tongue. ns, nonsignificant.

To further elucidate the differential responses of the anal and oral mucosae, we investigated the potential involvement of the PI3K/mTOR signaling pathway in the initiation and progression of squamous epithelial carcinogenesis in both sites. Given that the phenotypes observed in the anal and oral mucosae depend on E6/E7 expression and that the status of mTOR pathway activation remains unclear (19), we first examined the effects of PI3K pathway activation independently in each tissue. We employed a conditional murine knock-in model harboring a frequently reported activating mutation in PIK3C (28). The *Pik3ca*^H1047R^ mouse line expresses a constitutively active form of the p110 subunit of PIK3CA, generated by a histidine-to-arginine substitution at codon 104 (*Pik3ca*^H1047R^; ref. 28). Up to 2 months after TAM induction, K14-CreER^tam^/*Pik3ca*^H1047R/+^ (K14/PI3K) mice showed no overt phenotype (Fig. 4). However, histologic analysis revealed morphologic changes in the majority of tongues and anal mucosa compared with controls, including hyperplasia and mild dysplasia in the anal mucosa (3/3) and lingual epithelium (5/5; Fig. 4A, B, and D). At 4 months, these alterations persisted in both tissues (4/4; Fig. 4C, E, and F). Dysplasia was observed in 55% of lingual samples and hyperplasia and dysplasia in 100% of the anal mucosa (compatible with LSIL; Fig. 4G), whereas controls showed no changes. Both the anal and oral mucosae showed pS6 expression similar to that observed in TG-E6/E7 mice (Fig. 4H–J), reaching statistical significance compared with controls (Supplementary Fig. S5). Next, to examine whether the *Pik3ca* mutation cooperates with E6/E7 expression during anal or oral carcinogenesis, we bred the TG-E6/E7 mice with the *Pik3ca*^H1047R^ mouse line. C.M. showed normal weight gain, remained healthy, and displayed no evident phenotype during the 6-month follow-up. Necropsies performed at the indicated experimental time points revealed no macroscopic alterations in the anal and oral mucosae or skin (Fig. 5). Nonetheless, after 2 months, the anal mucosa of compound animals showed basal epithelial layer stratification and cellular atypia in all cases. Hyperplasia with hyperkeratosis and areas of hyperorthokeratosis/parakeratosis were observed (4/4; Fig. 5A and B). After 4 months of follow-up, in addition to the alterations observed at 2 months, epithelial differentiation defects were noted, including dyskeratosis in isolated cells and, at the tissue level, the presence of basal mitotic figures and an increased number of atypical nuclei in suprabasal layers. However, no cases were diagnosed as dysplasia (Fig. 5C). At 6 months, hyperplastic lesions persisted without progression (2/2). Moreover, in compound animals induced with TAM and treated with DOX for 2 months, followed by 2 months of withdrawal, the anal canal epithelium seemed histologically similar to compound anal mucosa after 2 months of follow-up; however, these animals did not develop mild dysplasia observed in K14/PI3K anal mucosa (2/2; Supplementary Fig. S3C). Therefore, anal mucosa of C.M. only developed lesions consistent with LSIL (Fig. 5H). With regard to the phenotype developed in the lingual epithelium of compound animals after 2 months of follow-up, dysplasia was diagnosed in the majority of the cases examined (5/6), ranging from mild to severe (Fig. 5D and E). Analysis of the phenotype after 4 months revealed that the dysplasia did not progress, although all cases exhibited moderate to severe dysplasia (3/3; Fig. 5F and G). At 6 months, dysplastic lesions persisted without progression (3/3). Tongues without prolonged expression of E6/E7 (tongues from C.M. induced with TAM followed by DOX treatment for 2 months and withdrawal of DOX and follow-up for 2 months) showed no hyperplastic or dysplastic alterations (2/2; Supplementary Fig. S3D). Overall, the compound tongue exhibited dysplasia in 83% of cases, whereas the remaining cases were hyperplasias (Fig. 5H). Lastly, both the anal canal and tongue from C.M. exhibited a significantly higher percentage and density of pS6-positive cells in the upper epithelial strata compared with control mice (Fig. 5I and J; Supplementary Fig. S5). Anal squamous lesions are commonly classified using the LSIL/HSIL system in accordance with current World Health Organization recommendations (36), whereas oral lesions are typically graded according to the degree of epithelial dysplasia (37), reflecting differences in established histopathologic frameworks between these anatomic sites. Finally, to formally compare the impact of the different signaling pathway manipulations across squamous epithelia from distinct anatomic sites, we reclassified the anal mucosa lesions into hyperplastic and dysplastic categories. Figure 6 summarizes the incidence of each lesion type in the anal mucosa and tongue epithelium across all experimental groups. An omnibus statistical analysis revealed a significant three-way interaction among anatomic location, pathway manipulation, and histological category (Fig. 6). *Post hoc* analyses showed the expected increase in premalignant lesions in the TG-E6/E7, K14/PI3K, and C.M. groups compared with controls, regardless of anatomic location. However, significant location-dependent differences emerged specifically in the TG-E6/E7 and compound groups; both manipulations produced a significantly higher incidence of dysplasia in the tongue compared with the anal mucosa. In contrast, PI3K pathway activation yielded comparable lesion profiles at both sites (Fisher exact test, *P* = 0.94; Fig. 6). Collectively, these findings demonstrate that identical signaling alterations elicit distinct premalignant phenotypes depending on the tissue context, even among squamous epithelia, highlighting the critical influence of the native epithelial microenvironment on oncogenic pathway activity.

## Discussion

High-risk HPV infections finely modulate epithelial homeostasis, establishing a new equilibrium between cell proliferation and differentiation. This new balance is crucial not only for viral persistence but also for the subsequent development of neoplastic changes. The infectious cycle of HPV is intricately linked to the differentiation program of keratinocytes (1, 10). Our results confirm that expression of HPV16 E6/E7 in epithelial compartments containing stem cells, both anal and oral, leads to the development of dysplastic changes within 2 months. Importantly, we demonstrate that the anatomic location—whether anal or oral—determines the severity of these lesions, even when the same viral oncoproteins are expressed.

Our model highlights the importance of tissue-specific factors in the early stages of carcinogenesis. Although HPV16 E6/E7 expression disrupted epithelial homeostasis in both anatomic sites, the incidence and grade of dysplasia varied significantly. The underlying tissue context, including the presence of stem cells within the epithelium, may play a role in modulating the carcinogenic process, particularly considering that our Cre-driver line targets both progenitor and differentiating cells (18). This observation underscores the necessity of considering the local microenvironment when studying HPV-related pathogenesis. Notably, previous animal models have shown that HPV E6/E7 expression in the anal canal does not lead to the development of premalignant changes (15, 38), whereas oral mucosa models tend to exhibit lower incidences of oral tumors (39). Our animal model system offers a new perspective by targeting both tissues simultaneously, allowing a direct comparison of HPV-induced carcinogenesis in two distinct epithelial environments.

The expression of HPV16 E6/E7 oncoproteins in the basal layer of epithelial tissues is a key event in the initiation of carcinogenesis. In our study, we demonstrate that HPV16 E6/E7 is expressed in a cellular compartment that includes epithelial stem cells committed to squamous differentiation. Using lineage tracing with the tdTomato reporter, we show that the E6/E7 oncoproteins disrupt epithelial differentiation by targeting a stem cell–containing niche. The expression of E6 in basal cells and some suprabasal cells, distributed in patches, indicates that these oncoproteins exert their effects on both progenitor and differentiating cells.

Our findings align with the hypothesis that the initiation of HPV-associated diseases requires infection not just of basal epithelial cells but more specifically of epithelial stem cells located within cutaneous or mucosal sites (40–42). This model suggests that stem cell populations are particularly vulnerable to HPV-induced transformation, which may explain the persistence and progression of dysplastic lesions in the anal and oral mucosae. Furthermore, our findings provide evidence that the proliferative phenotype associated with E6/E7 expression is directly linked to continuous expression of these oncoproteins, as cessation of DOX administration halted lesion progression, further supporting the idea that HPV-induced dysplasia is a dynamic process dependent on sustained activity of HPV oncoproteins.

To further investigate the molecular mechanisms driving HPV-induced carcinogenesis, we assessed the role of the PI3K/mTOR signaling pathway in lesion development. Although PI3K pathway activation alone did not induce malignant transformation and resulted in comparable premalignant alterations at both anatomic sites, its combination with E6/E7 expression produced marked tissue-specific differences in the incidence and severity of dysplastic lesions in the anal and oral mucosae. Specifically, lesions in the oral mucosa exhibited a higher incidence and more severe grade of dysplasia upon PI3K activation compared with the anal mucosa. This differential response could be due to the distinct microenvironments of the two tissues, suggesting that the presence of specific stem cell populations or other, yet to be identified, tissue-specific factors may influence the outcome of signaling pathway activation in each location.

Interestingly, combining PI3K activation with HPV16 E6/E7 expression did not accelerate malignant transformation at either site. However, it is worth noting that previous studies have reported that mutant forms of Pik3ca, when combined with HPV16 oncoproteins, promote earlier onset of anal carcinogenesis (38). In contrast to these findings, our results indicate that although PI3K activation enhanced lesion development, it was not sufficient to drive malignant transformation. Different transgenic models do not always yield fully consistent results, and such discrepancies often arise from the specific cellular compartment targeted by the driver mouse line employed. Although Shin and colleagues (38) relied on a constitutive overexpressing driver for E6/E7, our system provides temporal and tissue-restricted control through a dual inducible strategy for both transgenes. Our results suggest that other pathways, perhaps related to the microenvironment or alternative signaling cascades, may be involved in mediating the transformation process in HPV-associated cancers. In the context of HNSCC, it has been reported that mTOR is activated in many cases despite the absence of mutations in the PI3K pathway (43), indicating that alternative mechanisms may drive the activation of mTOR and contribute to the progression of these cancers. For instance, YAP/TEAD-mediated transcription has been recently shown to activate mTOR in oral epithelial cells through an AXL/EGFR signaling cascade (44), highlighting the complexity of signaling processes in the oral mucosa that our model may not entirely recapitulate.

Our study emphasizes the importance of evaluating the PI3K pathway in the correct tissue context, as the pathophysiologic outcomes of pathway activation can differ markedly between the anal and oral epithelia. Moreover, our findings suggest that the lack of malignant transformation in both tissues, despite PI3K pathway activation, may be due to the absence of additional oncogenic “hits” or the requirement for other molecular events, including the activation of alternative aberrant signaling mechanisms.

In summary, our study underscores the importance of tissue context in shaping the outcomes of HPV16-induced carcinogenesis. Identical signaling alterations, such as the expression of E6/E7 oncoproteins or the activation of the PI3K pathway, can lead to distinct premalignant outcomes depending on the local tissue microenvironment, at least in this model. Our findings further demonstrate that HPV16-induced dysplasia in the anal and oral mucosae could be shaped by tissue-specific factors, including the presence of epithelial stem cells, which must be carefully considered when studying HPV-associated cancers. Ultimately, our results emphasize the need to assess signaling dysregulation within its native epithelial context to better understand the complexity of HPV-related oncogenesis and to identify specific potential therapeutic targets in the near future.

## Supplementary Material

Supplementary Figure 1 Transgenic mouse models. Schematic representation of the spatiotemporally controlled expression of E6 and E7, along with PI3K-mediated activation.

Supplementary Figure 2 Schematic illustrating temporal control in TG-E6/E7 and compound mice.

Supplementary Figure 3 Histological Features of Anal and Oral Mucosa Following Inducible E6/E7 Expression and Withdrawal.

Supplementary Figure 4 DOX withdrawal reverts the proliferative phenotype in the Tg- E6/E7 anal mucosa

Supplementary Figure 5 Quantitative analysis of pS6 expression by immunohistochemistry in tongue and anal mucosa from control, TG-E6/E7, K14/PI3K, and compound mouse.

Supplementary Table 1 Primer sequences used for genotyping each of the mouse lines employed in this study.

## Data Availability

All data supporting the findings of this study are available within this article, Supplementary Files, or from the corresponding author upon reasonable request.
