# Supplementary material for: Tissue Context Shapes Distinct Premalignant Outcomes in an HPV16 E6/E7-Mutant Pik3ca Transgenic Mouse Model
Source: Cancer Res Commun. 2026 Jul 22;6(7):1750–61. doi: 10.1158/2767-9764.CRC-25-0789 (PMC13389264; doi:10.1158/2767-9764.CRC-25-0789)
Supplement: Supplementary Figure 1 — Transgenic mouse models. Schematic representation of the spatiotemporally controlled expression of E6 and E7, along with PI3K-mediated activation. [file crc-25-0789_supplementary_figure_1_suppsf1.pdf]

Supplementary Figure 1

Transgenic mouse models. Schematic representation of the spatiotemporally controlled expression of E6 and E7, along with PI3K-mediated activation.

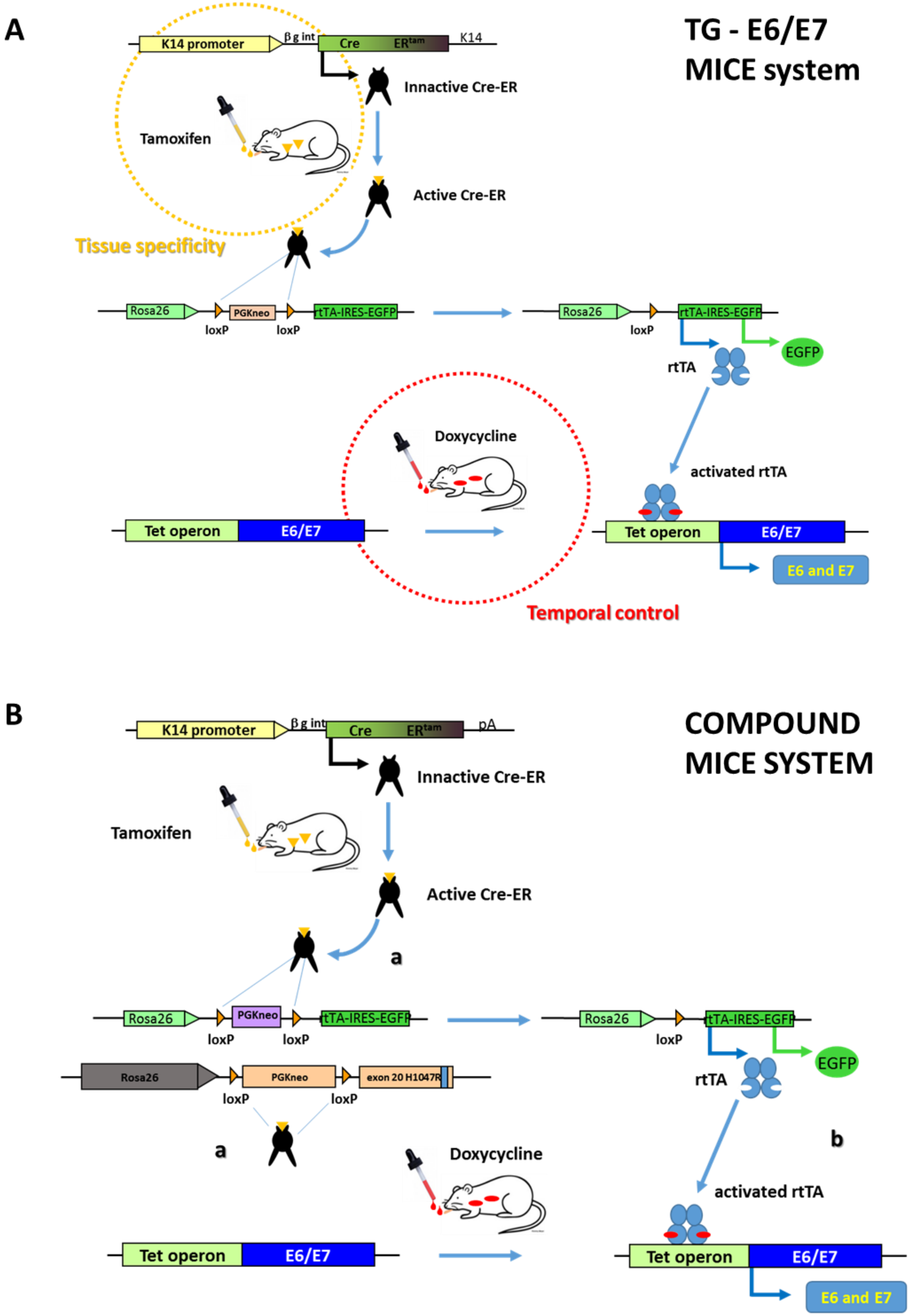

A- Schematic representation of the tissue-specific and temporal induction of viral oncoproteins E6 and E7 in anal and lingual mucosa. EGFP expression in the target tissue is first achieved in a Cre recombinase-dependent manner upon tamoxifen induction. Subsequently, doxycycline administration activates the Tet operon system, triggering E6 and E7 expression in K14-CreER^{tam} –targeted tissues.

B- Schematic representation of tissue-specific and temporal induction of viral oncoproteins E6 and E7 in the anal and lingual mucosa and PIK3ca^{H1047R}. EGFP and PI3K$^{H1047R\ is}$ are first expressed in the target tissue in a Cre recombinase-dependent manner upon tamoxifen induction (a). Doxycycline is then administered to activate the Tet operon system, inducing E6 and E7 expression in K14-CreER^{tam}–targeted tissues (b). Tissue-specific expression of the constitutively active PI3K variant is achieved via Cre-mediated excision of a stop element in the transgenic construct.
