# Supplementary material for: Tissue Context Shapes Distinct Premalignant Outcomes in an HPV16 E6/E7-Mutant Pik3ca Transgenic Mouse Model
Source: Cancer Res Commun. 2026 Jul 22;6(7):1750–61. doi: 10.1158/2767-9764.CRC-25-0789 (PMC13389264; doi:10.1158/2767-9764.CRC-25-0789)
Supplement: Supplementary Figure 2 — Schematic illustrating temporal control in TG-E6/E7 and compound mice. [file crc-25-0789_supplementary_figure_2_suppsf2.pdf]

Supplementary Figure 2.

Schematic illustrating temporal control in TG-E6/E7 and compound mice.

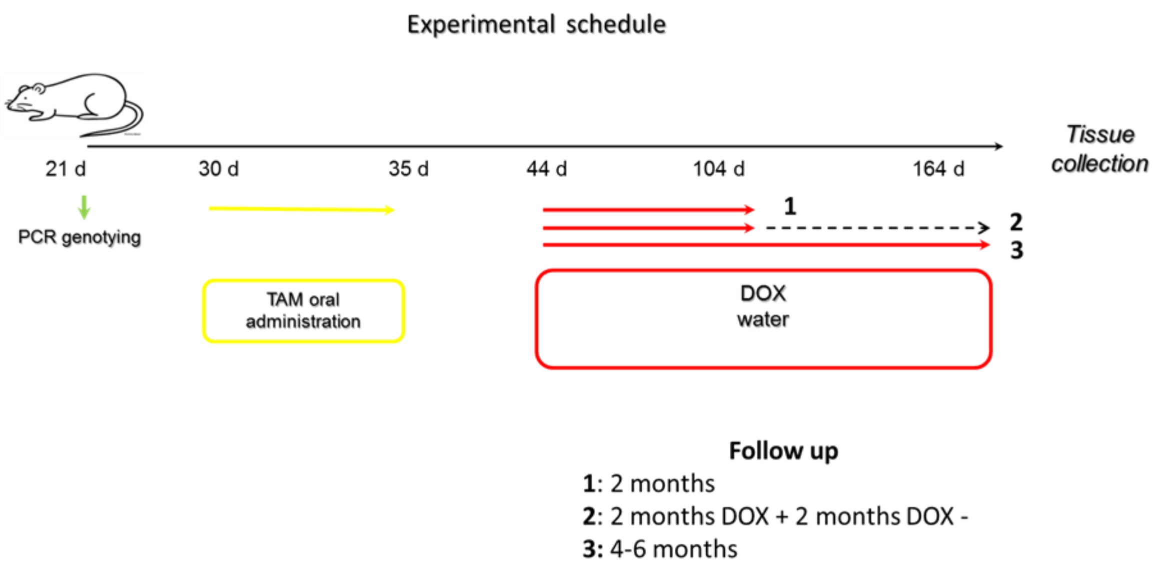

Temporal control in TG-E6/E7 and compound mice. Timeline of the general experimental procedure. Following genotyping and selection of the appropriate animals, tamoxifen is administered orally for 5 days. In a second, sequential stage, DOX is provided in the drinking water throughout the experimental period, according to the experimental scheme used (1-3).
