# Supplementary material for: Tissue Context Shapes Distinct Premalignant Outcomes in an HPV16 E6/E7-Mutant Pik3ca Transgenic Mouse Model
Source: Cancer Res Commun. 2026 Jul 22;6(7):1750–61. doi: 10.1158/2767-9764.CRC-25-0789 (PMC13389264; doi:10.1158/2767-9764.CRC-25-0789)
Supplement: Supplementary Figure 3 — Histological Features of Anal and Oral Mucosa Following Inducible E6/E7 Expression and Withdrawal. [file crc-25-0789_supplementary_figure_3_suppsf3.pdf]

Supplementary Figure 3.

Histological Features of Anal and Oral Mucosa Following Inducible E6/E7 Expression and Withdrawal.

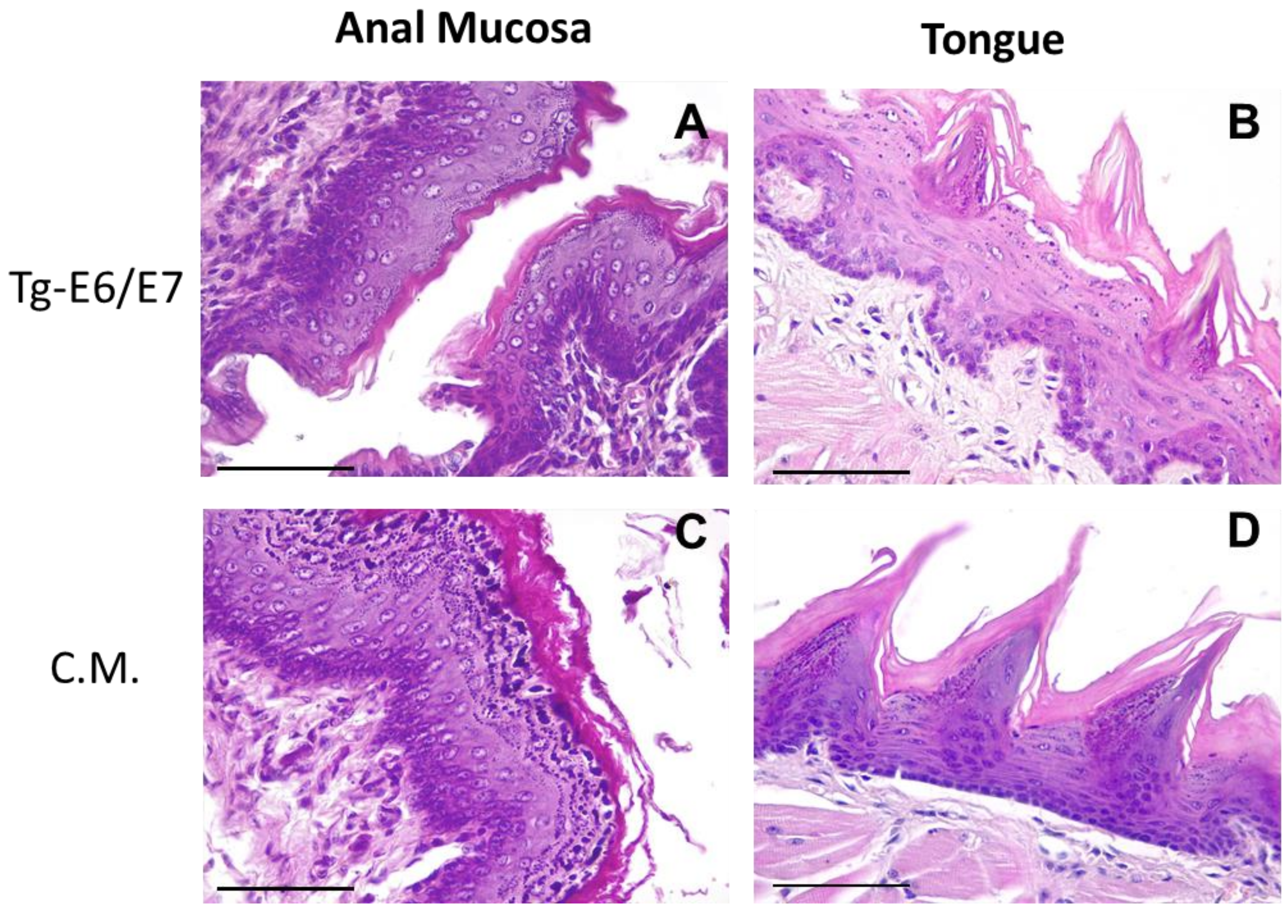

Histological features in AM and Tongue from TG-E6/E7 and CM mice that underwent TAM induction, two months of DOX treatment followed by a two-month withdrawal to assess the need for sustained expression. **A**-Tg-E6E7 anal mucosa shows hyperkeratosis and hypergranulosis. **B**- Tg-E6/E7 oral mucosa within normal limits and epithelial hyperkeratosis. **C**-Anal mucosa from compound mice. Anal mucosa lined by epithelium with compact hyperkeratosis and hypergranulosis, showing areas with basal stratification. **D**- Oral tongue mucosa from compound mice (CM) within normal limits. Scale bars: 50 μm.
