# Supplementary material for: Tissue Context Shapes Distinct Premalignant Outcomes in an HPV16 E6/E7-Mutant Pik3ca Transgenic Mouse Model
Source: Cancer Res Commun. 2026 Jul 22;6(7):1750–61. doi: 10.1158/2767-9764.CRC-25-0789 (PMC13389264; doi:10.1158/2767-9764.CRC-25-0789)
Supplement: Supplementary Figure 4 — DOX withdrawal reverts the proliferative phenotype in the Tg- E6/E7 anal mucosa [file crc-25-0789_supplementary_figure_4_suppsf4.pdf]

Supplementary Figure 4.

DOX withdrawal reverts the proliferative phenotype in the Tg- E6/E7 anal mucosa

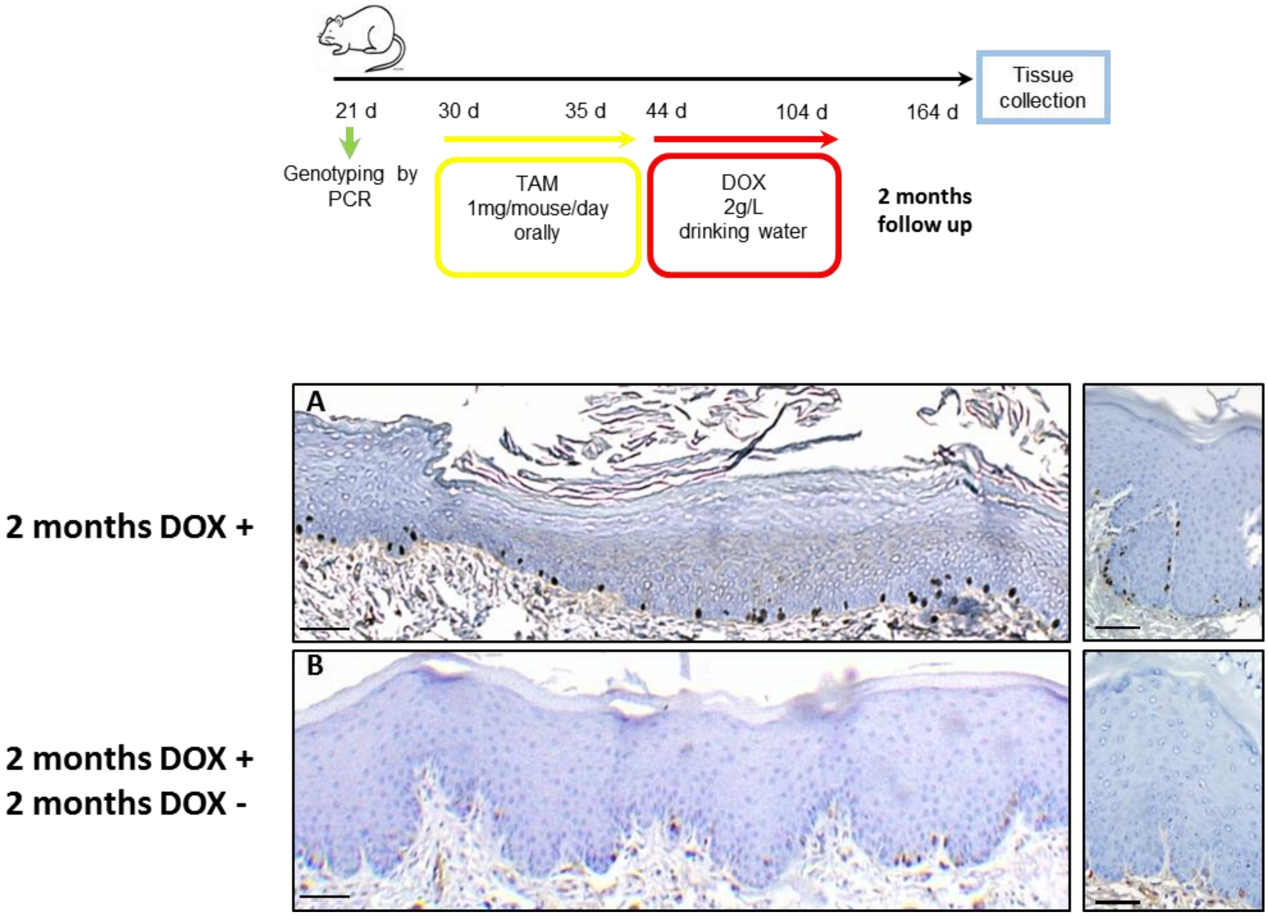

In the absence of DOX, the proliferative phenotype of the TG-E6/E7 anal mucosa is reversed. Qualitative assessment of cell proliferation was performed through BrdUrd incorporation. Representative images are shown. A- E6/E7 anal mucosa showing conspicuous positive labeling in the basal layer after two months of TG system induction. On the right, an image highlights an area of the basal layer with abundant basal stratum BrdUrd labeling. B- TG-E6/E7 anal mucosa from an animal treated with DOX for 2 months followed by 2 months without DOX. Note the reduction in basal BrdUrd-positive labeling. On the right, an image shows an example of anal mucosa from another TG-E6/E7 animal subjected to the 2 months DOX + 2 months without DOX protocol, displaying sparse BrdUrd-positive labeling in the basal layer. Scale bars: 50 μm.
