# Supplementary material for: Tissue Context Shapes Distinct Premalignant Outcomes in an HPV16 E6/E7-Mutant Pik3ca Transgenic Mouse Model
Source: Cancer Res Commun. 2026 Jul 22;6(7):1750–61. doi: 10.1158/2767-9764.CRC-25-0789 (PMC13389264; doi:10.1158/2767-9764.CRC-25-0789)
Supplement: Supplementary Figure 5 — Quantitative analysis of pS6 expression by immunohistochemistry in tongue and anal mucosa from control, TG-E6/E7, K14/PI3K, and compound mouse. [file crc-25-0789_supplementary_figure_5_suppsf5.pdf]

Supplementary Figure 5

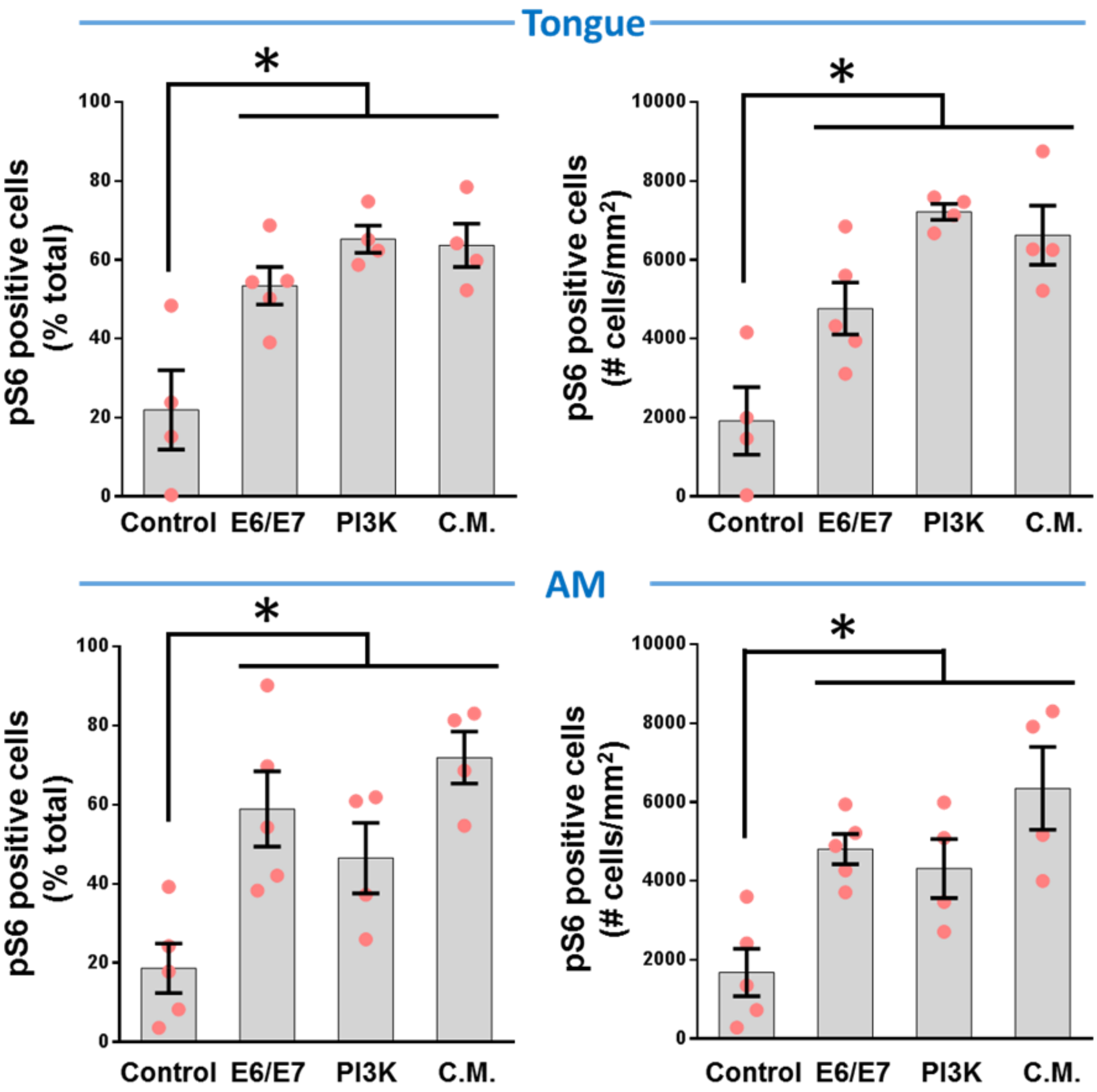

Quantitative analysis of pS6 expression by immunohistochemistry in tongue and anal mucosa (AM) from control, TG-E6/E7, K14/PI3K, and compound mouse (C.M.). The number of pS6-immunolabeled cells is expressed as the percentage of total epithelial cells identified by H&E counterstaining and as cell density per epithelial area, from 4–5 independent mice per group. Data are presented as mean ± SEM; each dot represents an individual animal. Statistical significance was assessed by one-way ANOVA (tongue: percentage, $F_{(3,13)} = 9.69$, $p = 0.0013$; density, $F_{(3,13)} = 12.0$, $p = 0.0005$; anal mucosa: percentage, $F(3,14) = 8.23$, $p = 0.0021$; density, $F(3,14) = 8.11$, $p = 0.0022$), followed by Holm–Šídák multiple comparisons post hoc tests. * $p < 0.05$ vs. control.
