# Supplementary material for: Tissue Context Shapes Distinct Premalignant Outcomes in an HPV16 E6/E7-Mutant Pik3ca Transgenic Mouse Model
Source: Cancer Res Commun. 2026 Jul 22;6(7):1750–61. doi: 10.1158/2767-9764.CRC-25-0789 (PMC13389264; doi:10.1158/2767-9764.CRC-25-0789)
Supplement: Supplementary Table 1 — Primer sequences used for genotyping each of the mouse lines employed in this study. [file crc-25-0789_supplementary_table_1_suppst1.pdf]

# Supplementary Table 1. Primer sequence for genotying.

| Amplified gene | Sequence (5’ to 3’) |
|---|---|
| K14-CreER$^{TAM}$ | Forward K14<br>5’-ATACCGGAGATCATGCAAGC-3’<br>Reverse K14<br>5’-AGGTGGACCTGATCATGGAG-3’<br><br>Forward IL2<br>5’-CTAGGCCACAGAATTGAAAGATCT-3’<br>Reverse IL2<br>5’-GTAGGTGGAAATTCTAGCATCATCC-3’ |
| Tet-E6/E7 | Forward 5’-<br>TGATCTCTACTGTTATGAGCAATTAAATG-3’<br><br>Reverse<br>5’-TGTCCGGTTCTGCCTGTCC-3’ |
| Rosa26-rtTA-IRES-EGFP-rtTA$^{flox}$ | Forward<br>5’-CTGGCTTCTGAGGACCG-3’<br><br>Reverse mutant<br>5’´-AGACTGCCTTGGGAAAAGCG-3’<br><br>Reverse wt<br>5’-AGCCTGCCCAGAAGACTCC-3’ |
| Pik3ca$^{H1047R}$ | Forward<br>5’-AAAGTCGCTCTGAGTTGTTAT-3’<br><br>Reverse mutant<br>5’-GCGAAGAGTTTGTCCTCAACC-3’<br><br>Reverse wt<br>5’-GGAGCGGGAGAAATGGATATG-3’ |
| Ai14 | Forward mutant<br>5’- CTGTTCCTGTACGGCATGG -3’<br>Reverse mutant<br>5’ - GGCATTAAAGCAGCGTATCC - 3’<br>Forward wt<br>5’ - AAGGGAGCTGCAGTGGAGTA - 3’<br>Reverse wt<br>5’ - CCGAAAATCTGTGGGAAGTC - 3’ |
